# Neurodegeneration of White and Gray Matter in the Hippocampus with FXTAS

**DOI:** 10.3390/ijms242417266

**Published:** 2023-12-08

**Authors:** Maryam Kargar, Randi J. Hagerman, Verónica Martínez-Cerdeño

**Affiliations:** 1 Institute for Pediatric Regenerative Medicine, Shriners Hospitals for Children, Sacramento, CA 95817, USA; 2 Department of Pathology and Laboratory Medicine, UC Davis School of Medicine, Sacramento, CA 95817, USA; 3 MIND Institute, UC Davis School of Medicine, Sacramento, CA 95817, USA; rjhagerman@ucdavis.edu

**Keywords:** pathology, hippocampus, FXTAS

## Abstract

Fragile X-associated tremor/ataxia syndrome (FXTAS) is a neurodegenerative disorder that affects older premutation carriers (55–200 CGG repeats) of the fragile X gene. Despite the high prevalence of the FXTAS disorder, neuropathology studies of individuals affected by FXTAS are limited. We performed hematoxylin and eosin (H&E) staining in the hippocampus of 26 FXTAS cases and analyzed the tissue microscopically. The major neuropathological characteristics were white matter disease, intranuclear inclusions in neurons and astrocytes, and neuron loss. Astrocytes contained more and larger inclusions than neurons. There was a negative correlation between age of death and CGG repeat length in cases over the age of 60. The number of astroglial inclusions (CA3 and dentate gyrus) and the number of CA3 neuronal inclusions increased with elevated CGG repeat length. In the two cases with a CGG repeat size less than 65, FXTAS intranuclear inclusions were not present in the hippocampus, while in the two cases with less than 70 (65–70) CGG repeat expansion, neurons and astrocytes with inclusions were occasionally identified in the CA1 sub-region. These findings add hippocampus neuropathology to the previously reported changes in other areas of the brain in FXTAS patients, with implications for understanding FXTAS pathogenesis.

## 1. Introduction

Fragile X-associated tremor/ataxia syndrome (FXTAS) is an adult-onset neurodegenerative disorder caused by a CGG repeat expansion (55–200 repeats, premutation) within the 5′ UTR of the fragile X gene (fragile X messenger ribonucleoprotein 1 gene, *FMR1*) [1,2,3]. The *FMR1* protein (fragile X messenger ribonucleotide 1 protein, FMRP) is a critical factor in gene expression and translation regulation of many mRNAs that are involved in the development and function of neuronal synaptic connections [4,5]. The *FMR1* gene is highly polymorphic in the general population, ranging from 6 to 55 triplets. CGG repeats of more than 200 is a full mutation (FM) and produce the neurodevelopmental condition Fragile X Syndrome (FXS). In FM, there is a loss of FMR1 messenger and protein. *FMR1* with a shorter expansion, between 55 and 200 CGG repeats, is referred to as a premutation (PM) of the fragile X gene, and carriers of the fragile X premutation are at risk of developing FXTAS. Unlike the FM, the alleles of the PM range are functional, enabling the induction of an excess of *FMR1* messenger RNA and normal or decreased FMRP [2,6,7,8,9,10,11]. The prevalence of premutation carriers is estimated at approximately 1 per 400 males (Ms) and 1 per 291 females (Fs). However, female premutation carriers present with lower rates of FXTAS but more frequent psychiatric problems compared with males with the premutation. FXTAS impacts nearly 16.5% of the women carriers of the *FMR1* premutation and about 45.5% of the men carriers over the age of 50 years old. This gender-associated difference may come from the protective effect of the second X chromosome in the somatic cells of female patients with FXTAS [12,13,14,15,16,17]. The major clinical phenotypes of FXTAS include intention or progressive kinetic tremor, cerebellar gait ataxia, and neurocognitive implications. Other features commonly observed in FXTAS are parkinsonism, lower extremity neuropathy, psychiatric involvement (including anxiety, disinhibition, depression, and apathy), autonomic dysfunction (namely orthostatic hypotension, impotence, and eventually urine and stool incontinence), dementia, and, in some individuals, early death. The end stages of the disorder are characterized by laryngeal muscle weakness, and death is usually caused by dysphagia and pulmonary infections. The life expectancy of FXTAS patients has been reported to be variable, ranging from 5 to 25 years after diagnosis [18,19,20,21,22]. In addition to the clinical signs, radiological features contribute to the diagnosis of FXTAS. Radiologic evaluations using magnetic resonance imaging (MRI) have identified white matter disease, global brain atrophy, and ventricular enlargement [18,23,24,25]. The neuropathological changes of FXTAS include dystrophic white matter (demyelination in the white matter), the presence of intranuclear inclusions in both neurons and astroglial cells, iron deposition, and microbleeding [21,26,27,28,29]. Despite the importance of this disorder as a high personal and economic burden for society, the number of pathology reports on people involved with FXTAS is currently very limited. We herein evaluated the neuropathological changes in the postmortem hippocampus of 26 FXTAS patients and 9 controls. We provide an explanation of hippocampal neuropathology in FXTAS.

## 2. Results

### 2.1. Neuropathology

We collected brain tissue from the hippocampal formation of 26 cases diagnosed with FXTAS (6 females (Fs), 19 males (Ms), and one unknown), and 9 control cases (6 Fs, 3 Ms). FXTAS was diagnosed based on the observations of the neurological assessment. The average age at time of death was 73.37 years with a range of 34–89 years for the FXTAS cases and of 68.1 years with a range of 53–86 years for the control cases. FXTAS brains were presented with an average postmortem interval (PMI) of 45 h, while the control cases had an average PMI of 38.2 h. The average number of CGG triplet repeats for FXTAS subjects was 81.59, ranging from 57–122 repeats. The number of CGG repeats for three FXTAS cases was not available. Age, PMI, and molecular data (CGG length number) of both FXTAS and control groups are listed in Table 1. All of the cases (26) were diagnosed with the presence of intranuclear inclusions in astrocytes and neurons within different brain areas that were evident with ubiquitin and H&E stains, consistent with a diagnosis of FXTAS [23]. 

On macroscopic examination of 26 patients with FXTAS, 13 cases had brain atrophy based on brain weight, and one brain had a normal weight. Of these 13 brains with atrophy, hippocampal tissue in one case was unremarkable, and three cases (3 of 26: 11.53%) revealed mild to moderate hippocampal atrophy. The hippocampus of these three subjects with FXTAS was generally smaller compared with the control hippocampus. The macroscopic information for the rest of the subjects was not available. The hippocampus tissue in the control group was grossly normal.

**Figure 1:**
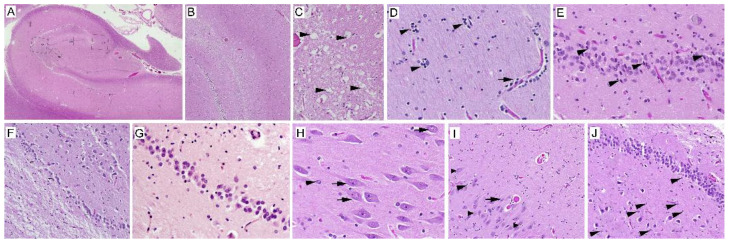
(**A**) Hippocampus of a control case without significant pathological findings. Hippocampal subfields are marked: the cornu ammonis (CA) and the dentate gyrus (DG). The squares delineate the two areas of CA (CA1 and 3) and two of DG (DG1 and 2) analyzed in this study (H&E, ×360). (**B**–**J**) Hippocampus neuropathological characteristics of human FXTAS in H&E. (**B**) The FXTAS hippocampal white matter depicts gradations of severe spongiosis, indicative of vacuolar degeneration and parenchymal pallor in subcortical white (H&E, ×36). (**C**) Higher power of vacuolar changes (arrowheads) (H&E, ×360). (**D**) The FXTAS hippocampus presents disseminated white matter gliosis (arrowheads) and PVC (arrows). (**E**) Necrotic neurons within the granular layer of a hippocampus with FXTAS, shrunken eosinophilic cytoplasm, and pyknotic or karyorrhectic nuclei (arrowheads). (**F**) In FXTAS, the hippocampus reveals severe neuron loss and spongiosis (**D**–**F**; H&E, ×360). (**G**) Higher power of subfigure (**F**) (H&E, ×720). (**H**) Eosinophilic intranuclear inclusions in pyramidal cells (arrows) and an astroglial cell (arrowhead) in the FXTAS hippocampus. (**I**) There are two types of intranuclear inclusions in CA3 pyramidal cells. Inclusions with a white halo that are more eosinophilic (thick arrowheads) and some inclusions without vacuole (thin arrowhead). An inclusion-bearing astrocyte in the same area (arrow). (**J**) Intranuclear inclusions in granule cells of the dentate gyrus of case 11 (arrowheads) (**H**–**J**; H&E, ×720).

**Figure 2:**
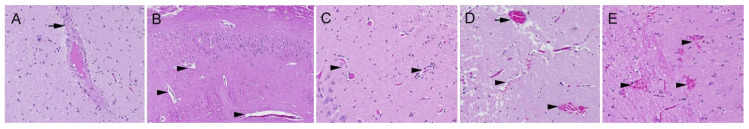
Vascular changes of the hippocampus in FXTAS include (**A**) thickening and hyalinized walls (arrow). (**B**) perivascular clearing (arrowheads). (**C**) Perivascular cuffing (PVC) (arrowheads). (**D**) Hypertension (arrow) and (**D**,**E**) microhemorrhages (arrowheads) (H&E, ×360).

A microscopic postmortem examination was performed in all cases. The most prominent neuropathological feature identified in the FXTAS hippocampus was the presence of intranuclear inclusions in both neurons and astrocytes throughout the tissue. White matter change, characterized by vacuolar degeneration (spongiosis) and white matter myelin loss, was the second neuropathological lesion in the hippocampus with FXTAS. Ten of 26 FXTAS cases (38.46%) presented with white matter changes in the hippocampus. Six of 26 cases (23.07%) showed mild degeneration (up to 39% of cells were degenerated), and three of 26 FXTAS cases (11.53%) demonstrated moderate degeneration (40% to 69% degeneration). Severe spongiosis was observed in only one case (3.84%), with most or all (70 to 100%) tissue degenerated (Figure 1B,C). Another common histopathological observation in FXTAS was diffuse white matter gliosis, identified in most of the cases (84.6% of cases) (Figure 1D). The hippocampus in one of 26 (3.84%) patients was affected by severe, 4 of 26 (15.38%) by moderate, and 17 of 26 (69.38%) by mild gliosis changes. In addition, necrotic neurons with pyknosis, karyorrhexis, karyolysis, and cytoplasmic eosinophilia were observed in 6 of 26 FXTAS subjects (23%) (Figure 1E). Intraparenchymal blood vessels had thickening and hyalinization of the walls (with a severity of 7.69% moderate and 65.38% mild vascular hyalinosis), perivascular clearing as widened perivascular spaces (7.69% moderate and 73.07% mild), and perivascular cuffing (PVC)—infiltration of inflammatory cells around the brain vessels (19.23% moderate and 73.07% mild). Furthermore, there were red blood cells in the parenchyma referred to as microhemorrhages (3.84% moderate and 30.76% mild) that may be indicative of hypertension (Figure 2A–E). Hemosiderin deposits (hemosiderosis) around the vessels or in the parenchyma (34.61% mild) were present in the hippocampus in FXTAS cases. The remaining FXTAS cases were considered microscopically normal. The hippocampus in the control group revealed no lesions at the microscopic level. Additionally, three of the patients (case numbers 3, 16, and 20) were characterized by Alzheimer’s disease (AD) neuropathological changes, consisting of intracellular neurofibrillary tangles and extracellular amyloid plaques [28]. The neuropathological features in the post-mortem hippocampus of FXTAS subjects can be found in Tables S1–S3.

### 2.2. Quantitative Analysis of the Number of Neurons and Astrocytes

**Figure 3:**
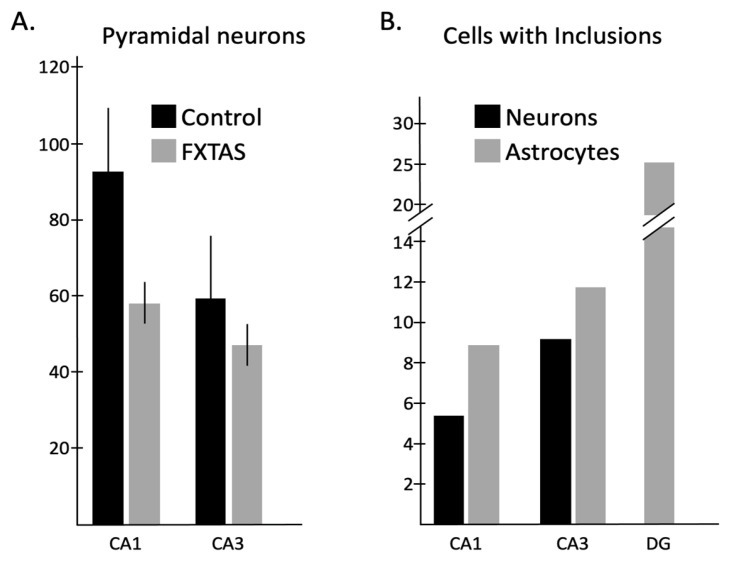
Cells (neurons and astrocytes) and inclusion number analysis of the FXTAS hippocampus. (**A**) The number of pyramidal neurons in both CA1 and CA3 areas of FXTAS cases (58.34 ± 27.37 and 47.4 ± 16.28, respectively) significantly declined by 34% and 11.9% compared to the control (92.87 ± 42.34 and 59.3 ± 15.2) (*p* < 0.05). (**B**) The number of astrocytic inclusions in DG (25.49%) was higher than in CA1 or CA3 (8.78% and 11.02%, respectively). In the CA area, the number of astrocytic inclusions was higher than neuronal inclusions, and also, the number of inclusions (both neuronal and astrocytic) in the CA3 area was higher than in CA1 (the percentage of neurons and astrocytes with inclusions for CA1 was 5.47% and 8.78%, and for CA3 it was 8.85% and 11.02%, respectively).

We quantified the total number of neurons and astroglial cells in four hippocampal sub-regions, consisting of CA1, CA3, DG1, and DG2, within regions of interest (ROIs) of both the control and FXTAS groups, as seen in Table 2 and Table 3. We then assessed the number of necrotic neurons (total numbers and percentage) using the same ROIs in CA1 and 3 and DG1 and 2 (Table S4, Section 4.5 for more detail). We found that the number of pyramidal neurons in both CA1 and CA3 areas of FXTAS cases (58.34 ± 27.37 and 47.4 ± 16.28, respectively) significantly decreased by 34% and 11.9% compared to the control cases (92.87 ± 42.34 and 59.3 ± 15.2; *p* < 0.05) (Figure 3A). Based on the average number of CA1 and CA3 pyramidal neurons in the control, the cell dropout in FXTAS varied in degree of severity, classified into four states: normal or no change (1), mild (2), moderate (3), and severe (4) (Figure 1F,G and Table S2). It should be noted that the severity of neuron loss in the females was less than in the male FXTAS subjects. We found more necrotic neurons in CA than in the DG area. As evident from Table S4, the average percentage of necrotic neurons for CA1 and CA3 was 9.1% (range 0–47.2%) and 8.4% (range 0–25%), respectively, whereas 3.1% (range 0–28%) and 2.1% (range 0–8.88%) of DG1 and DG2 of neurons were necrotic. No significant difference was observed in the percentage of necrotic neurons between the female and male FXTAS patients.

### 2.3. Quantitative Analysis of the Number of Neurons and Astrocytes with Intranuclear Inclusions

The intranuclear neuronal and astroglial inclusions throughout the hippocampus that are characteristic of FXTAS were single, discrete, round/ovoid-shaped, hyaline-appearing, eosinophilic, and stained with ubiquitin (Figure 1H–J). Intranuclear inclusions were located in the vicinity of the nucleolus, and in astrocytes they were massive, up to 80% of the size of the nucleus, while in neurons they were smaller and about the size of the nucleolus [23]. We observed two types of intranuclear inclusion bodies in astrocytes and neurons (CA and DG). Although most of the inclusions were eosinophilic, with a vacuole presenting as a white halo around, a few inclusions were less eosinophilic and did not have a vacuole (Figure 1I). Both male and female FXTAS cases revealed intranuclear inclusions with similar appearances in neurons and astrocytes. 

We counted the number and percentage of neurons with inclusions within the ROIs of CA1 and 3, and the number and percentage of astrocytes bearing inclusion within the ROIs of CA1 and 3 and DG1 and 2, detailed in Table 3 and Table 4. We identified neuronal inclusions in the DG granule cells but did not quantify them. From more than 4800 neurons and astrocytes quantified, all 517 inclusions scored were exclusively intranuclear; that is, no inclusions were detected in the cytoplasm of neural cells in tissue from the control or FXTAS cases. Moreover, we did not observe intranuclear inclusions in neurons or astrocytes in any of the control cases. As seen in Table 4, the percentages of cells with intranuclear inclusions were as follows: 6.95% for CA (1 and 3) pyramidal neurons (188 neurons with inclusions of 2703 neurons); 5.47% for CA1 (83 neurons with inclusions of 1517 neurons); and 8.85% for CA3 (105 neurons with inclusions of 1186 neurons). The percentages of cells with intranuclear inclusions were as follows: 10.08% for CA (1 and 3) astrocytes (149 astrocytes with inclusion of 1477 astrocytes); 8.78% for CA1 (54 astrocytes with inclusion of 615 astrocytes); 11.02% for CA3 (95 astrocytes with inclusion of 862 astrocytes); and 25.49% for DG (1 and 2) astrocytes (180 astrocytes with inclusion of 706 astrocytes). We found that the number of astrocytes with inclusion was higher than neurons with inclusion, as the percentage of astrocytes with inclusion for CA was 10.08% and only 6.95% of CA neurons possessed intranuclear inclusions. In spite of this outcome, there was a great deal of variability among cases. Moreover, the number of inclusion-bearing astrocytes was higher in DG compared to CA; the percentage of astrocytes with inclusion in CA was 10.08%, and 25.49% of DG astrocytes contained inclusion (Figure 3B). In addition, as identified in Table 4, the number of intranuclear inclusions (within astrocytes and particularly neurons) in CA3 was higher than in the CA1 area. While the percentage of neurons and astrocytes with inclusions for CA1 was 5.47% and 8.78%, respectively, 8.85% and 11.02% of CA3 neurons and astrocytes showed inclusions, respectively (Figure 3B). Although we found that the percentage of neurons and astrocytes with inclusions in the male with FXTAS was greater than the female FXTAS cases in the four sub-regions, only the percentage of DG2 astrocytes with inclusions in the Ms (24.16 ± 15.56) was significantly higher than in the Fs (9.43 ± 10.40) (*p* < 0.05).

### 2.4. Correlation Analysis 

We performed correlation analysis between histological findings and the number of CGG repeats, age, cell numbers, and inclusion numbers. There was a negative correlation between age of death and CGG repeat length, which was not statistically significant (*p* ≥ 0.05). However, by removing the three cases under age 60 according to the age of the first signs of FXTAS (60), the decrease in age of death was associated significantly with increasing CGG repeat length (*p* = 0.008). The most striking correlation was between the number of CGG repeats and the percentage of astrocytes or neurons with inclusion in CA3 (*p* = 0.048 and *p* = 0.000, respectively). As identified in Table 3, the two patients with the lowest CGG repeat expansion (less than 65), case number 4 (57 CGG repeats) and case number 16 (63 CGG repeats), did not show intranuclear inclusions in the hippocampus sub-regions, and two other subjects with low (less than 70; 65 to 70) CGG repeat length, case number 12 (66 CGG repeats) and case number 13 (67 CGG), only presented with rare FXTAS inclusions in some of the hippocampus sub-regions. Furthermore, there was a statistically significant correlation between CGG repeat number and the percentage of inclusion-bearing astrocytes in DG1 (*p* = 0.009) and the percentage of inclusion-bearing astrocytes in DG2 (*p* = 0.004). The correlation between the number of DG1 astrocytes and the percentage of DG1 astrocytes with inclusions was significant (*p* = 0.021). There was a significantly positive correlation between the percentage of DG1 astrocytes with inclusions and the percentage of DG2 astrocytes containing inclusions (*p* = 0.014). There were correlations between the percent of inclusions (neurons or astrocytes) and age of death, though not statistically significant (*p* ≥ 0.05). Case number 20 with 75 CGG repeats was an interesting FXTAS subject with AD criteria, with the maximum percentage of neurons with inclusions in CA1 and CA3, and the maximum percentage of astrocyte-bearing inclusions in CA1, but with the lowest number of neurons in CA1 compared to other FXTAS subjects in the current study. Significant positive correlations were found between the severity of microhemorrhages and three aspects of vascular changes, including PVC (*p* = 0.008), hypertension (*p* = 0.025), and wall thickness (*p* = 0.004). Moreover, PVC degree and age of death were found to be significantly correlated (*p* = 0.027). 

## 3. Discussion

This study provides an insight into hippocampal neuropathology that is in accordance with the past identified changes in FXTAS patients [23,28,30,31].

We found brain atrophy (brain volume loss) in almost all FXTAS cases, as previously reported [18]. The most prominent neuropathological feature was white matter involvement of the hippocampus in close to half of the subjects, in line with mild to moderate hippocampal atrophy. In a few past studies, premutation carriers have shown volume loss in the hippocampus and the amygdala-hippocampal complex [32,33]. Likewise, the hippocampus of female carriers (with or without FXTAS) was comparatively smaller than that of males with the premutation in a prior study [24]. This outcome is in contrast with other reports that described no difference in hippocampal volume of males with the FMR1 premutation [24,34]. The areas of white matter change were depicted with variable degrees of vacuolar degeneration (spongiosis), which corresponds to several reports of spongiosis in the cerebral white matter of subjects with FXTAS [23,35,36]. Further MRI examinations confirmed white matter abnormalities in some brain parts consisting of the hippocampal-amygdala complex among individuals with the premutation [31,37]

Moreover, mild to moderate microhemorrhages (indicative of hypertension) and mild hemosiderosis were observed in the hippocampus of some FXTAS cases, as previously reported [23]. More than half of FXTAS patients showed vessels with wall hyalinosis, widened perivascular spaces, and perivascular cuffing. These data are in line with the vascular changes in the post-mortem brains of people with FXTAS seen by Greco et al. [23], who stated perivascular parenchymal rarefaction was not observed along with these vascular changes. 

In addition to the involvement of white matter and vascular changes, the hippocampus neuropathology of FXTAS comprises neuron loss (gray matter changes/atrophy), gliosis, and intranuclear inclusion bodies.

### 3.1. Neuron Loss

There was a considerable pyramidal neuronal loss in the CA1 and CA3 sub-regions that varied from mild to severe as compared to the controls. These results are in agreement with other studies [23,30] that demonstrated diffuse gray-matter density loss, especially in the limbic regions, amygdala, and hippocampus, and suggest that the hippocampal atrophy in FXTAS might be due to neuron dropout and vacuolar degeneration. Moreover, fragile X premutation carriers presented low gray matter density in the amygdala-hippocampal complex [31,32]. Despite the fact that hippocampal neuron loss in CA1 is attributable to different etiologies, this change should be considered an objective criterion along with other hippocampus lesions for differential diagnosis. For instance, hippocampal sclerosis of aging is neuropathologically demonstrated by severe neuron loss within the CA1 area and the frequent presence of TDP-43 (transactive response DNA-binding protein of 43 kDa) inclusions [38,39,40,41].

### 3.2. Intranuclear Inclusions

The main postmortem pathological trait of FXTAS is intranuclear inclusions in neurons and astrocytes. In comparison with other brain regions, the hippocampus has the highest percentage of intranuclear inclusions in FXTAS patients. This may be attributable to the significant role of the limbic system in FXTAS clinical symptomology and also to the susceptibility of this system to RNA toxicity from the premutation state because the transcription rate of the FMR1 mutation in this region is higher than in other areas of the brain [23,35]. Indeed, the hippocampus has been identified with a high rate of FMR1 transcription during normal fetal development and high expression rates of FMR1 mRNA in adults, suggesting that the hippocampus may be especially affected by FXTAS [42]. Doxycycline-inducible mouse models that express RNA bearing an expanded CGG repeat are generated to induce FXTAS. Accordingly, the inducible mice with CGG repeat expansion of 90 CGGs have exhibited the hallmark intranuclear inclusions of FXTAS in the brain, with a high rate in the hippocampus and cerebellum [43].

We found that intranuclear inclusions in both neurons and astrocytes were distributed within different sub-regions of the hippocampus. The intranuclear inclusions were solitary, well-delineated, eosinophilic, distinct from the nucleolus, and smaller and fewer in neurons than the inclusions in astrocytes, according to past reports [23,28,30,35]. Although most of the neuronal and astroglial inclusions (CA and DG) were surrounded by a vacuole (a white/clear halo), a few were less eosinophilic and without a vacuole. Even though this is consistent with prior work reporting that the astrocytic inclusions are frequently enclosed by a clear halo, this could be a consequence of an artifact of tissue preparation [19,23]. The inclusion appearance in the male was the same as in the female FXTAS subjects. No cytoplasmic inclusions were detected in hippocampus tissue from FXTAS cases [14]. By contrast, the Drosophila model of FXTAS, the fly model of the premutation, contains a substantial number of inclusions within the cytoplasm [44]. We revealed that the number of intranuclear inclusions in pyramidal neurons of CA3 was numerous and greater than in CA1, conforming to a recent study by Sacino et al. [45], who reported high intranuclear inclusion bodies in CA3 pyramidal neurons of a 65-year-old male with FXTAS. Just as in Greco et al. [23], our data showed the percentage of inclusion-containing astrocytes in the dentate gyrus (DG1 and 2) was more than 25%. Conversely, there are two reports with an estimate of 11.56% and 6.35% DG astrocytes with inclusion by Tassone et al. [28] and Pretto et al. [29], respectively, and in another report, the inclusion-bearing astrocytes in the DG area were fewer than 2% [27]. As reported by Tassone et al. [28], our data identified that the percentage of astrocytic inclusions in CA1 was nearly 9%. Overall, our data are in accordance with other reports [23,28,29] indicating more astrocytes with inclusion in the DG sub-region than in the CA1 sub-region, and that in CA1, the number of astrocytic inclusions was higher than the neuronal inclusions. In contrast, another report outlined that there were more inclusions in astrocytes than in neurons of divergent hippocampal sub-regions, except for the CA1 area, proposing that it could be due to a great deal of inter-subject variability [23]. In this report, a similar number of intranuclear inclusions in neurons of each hippocampal sub-region was detected in both females and males affected by FXTAS, corroborating the research of Tassone et al. [28]. However, we found that the number of astrocytic inclusions in the DG2 area in the female was significantly fewer than in the male. This finding is in agreement with other studies revealing the number of astrocytes bearing inclusions was lower in female than in male FXTAS cases [23,28]. However, in two past studies, females with FXTAS contained fewer intranuclear inclusions in both neurons and astrocytes, which may be due to the effects of the extra X chromosome in the female subjects [17,23]. A pattern of advanced pathology in the hippocampus has been milder in FXTAS females than male patients [23]. Accordingly, the women in our current work exhibited milder neuropathological lesions, particularly the severity of pyramidal cell loss and spongiosis, compared to the males with FXTAS. 

### 3.3. Correlations

We found a negative correlation between the age of death and the length of the CGG repeat in patients over the age of 60, in accordance with other work [23,30]. This is concordant with a previous study by Greco et al. [23] demonstrating that the mean age of the first signs of FXTAS is in the early 60s. Generally, CGG repeat size has a positive correlation with pathological involvement, namely CNS atrophy, white matter change, and the formation of intranuclear inclusions [23]. Most patients with a larger CGG repeat expansion showed atrophy of hippocampal white and gray matter, in line with Moore and colleagues [31], who indicated a correlation between a higher CGG repeat number and reduced gray matter density. Contrary to the previous findings in FXTAS patients [23], we detected that the correlation between the percent of intranuclear inclusions (in both neurons and astrocytes) and the age at death was not significant. Older CGG ‘Knock-in’ (KI) mice revealed an increase in the number and size of FXTAS intranuclear inclusions in neurons over the lifetime compared with younger mice [46]. Interestingly, there was a positive correlation between the number of inclusions in astrocytes (of CA3, DG1, and DG2) and in neurons (of CA3) and the CGG repeat length. This finding corroborates other data in FXTAS stating that the number of cells with intranuclear inclusion increases with elevated CGG repeat size [23]. As a result, in our study, the subjects with the smallest CGG repeat expansion had no FXTAS intranuclear inclusions in the hippocampus (case numbers 4 and 16 with 57 and 63 CGG repeats, respectively); this is consistent with what has been found in previous publications [23,47]. The case with the fewest CGG repetitions (57 CGG repeats) was a young male premutation carrier (53 years old) that did not have inclusions in his hippocampus but did in other brain regions. The hippocampus of this patient presented with severe spongiosis, mild to moderate pyramidal cell loss, and evidence of severe diffuse acute cerebral ischemic damage. Spongiosis and neurodegenerative changes may have resulted from cerebral ischemic changes. The patient with the second-smallest CGG repeat number (63 CGG repeats), an older premutation carrier (80-year-old woman), had little evidence of FXTAS neuropathology containing mild pyramidal cell loss, with minimal evidence of hippocampus white matter disease and no inclusions in the hippocampus sub-regions, but did in Purkinje cells of the cerebellum. This woman presented with Alzheimer’s disease criteria comprising prominent granulovacuolar degeneration, scattered amyloid plaques, and neurofibrillary tangles in CA1. 

In a past study of male FXTAS subjects, an older premutation carrier with the lowest CGG repeat number (65 CGG repeats) was diagnosed with only rare inclusions seen in some of the brain areas. This man displayed a mild neurological phenotype characteristic of FXTAS but no evidence of cerebral white matter disease or intranuclear inclusions in his hippocampus [23]. A similar pattern was obtained in the analysis by Martínez-Cerdeño et al. [47], who reported from 40 brains of FXTAS carriers that two cases with repeat size in the 60s had no FXTAS inclusions, including a man (65 CGG repeats) and a woman (66 CGG repeats). The other two patients in our survey with less than 70 CGG repeat length (65–70), case numbers 12 and 13, with 66 and 67 CGG repeats, respectively, had rare intranuclear inclusions in CA1 neuronal and astroglial cells. It has been hypothesized that some additional factors, such as genetic or environmental factors, may predispose to the development of FXTAS in a person with a low CGG repeat number of less than 70 CGG repeats (with repeats in the 60s) [23,47]. Further research is needed to completely assess and explain the impact of low-end premutation alleles on FXTAS development and inclusion information. Three subjects (two women and a man) presented with AD neuropathological changes, confirming a past neuropathological study [28] that reported the co-occurrence of AD in people involved with FXTAS. 

## 4. Materials and Methods

### 4.1. Sample Collection

Postmortem hippocampal samples from 26 FXTAS and 9 control cases were obtained from the FXS/FXTAS brain repository at the University of California, Davis, a node of the Hispano-American Brain Bank on Neurodevelopmental Disorders (CENE). All tissue samples were obtained through consented autopsies with institutional review board approval, in accordance with the UC Davis IRB protocol. The controls, who matched the age range of FXTAS cases, lacked any significant neurological history at the time of death and were free of gross neuropathological features. Individuals with FXTAS had neurological symptoms for many years before death and were diagnosed based on the presence of FXTAS-related clinical signs, namely progressive kinetic tremor, cerebellar gait ataxia, peripheral neuropathy, psychiatric involvement, parkinsonism, memory and executive function deficits, and autonomic dysfunction. The diagnosis of FXTAS was confirmed by the presence of positive ubiquitin intranuclear inclusions in neurons and astrocytes in the postmortem brain. More information about case characteristics is summarized in Table 1.

### 4.2. H&E Staining

The hippocampus tissue of all FXTAS and control cases was blocked and coronally sectioned. Samples were fixed in 10% buffered formalin, followed by paraffin embedding of representative samples, and sectioned at 10 μm. Then, sections were stained with hematoxylin and eosin (H&E), dehydrated with ethanol, and cleared with xylene before being cover-slipped with Permount (Fisher, Pittsburg, PA, USA). 

### 4.3. Immunostaining

Antigen retrieval was performed with 1× diva solution (heat-induced epitope retrieval [HIER] buffer; Biocare Medical, Concord, CA, USA) at 80–110 °C (8 min), followed by pretreatment for 7 min with 3% hydrogen peroxide (Fisher, CA, USA), 10% donkey serum, and 0.025% Tween 20 (Fisher, CA, USA) for 1 h. Tissue samples were subsequently incubated with rabbit anti-ubiquitin primary antibody (1:200, Dako, Glostrup, Denmark) diluted in 10% donkey serum and 0.0025% Tween 20 (4 °C, 15 h). After rinsing, samples were incubated in the donkey anti-rabbit secondary antibody diluted as above for 1 h at room temperature (1:100, Jackson Immunoresearch, West Grove, PA, USA). Samples were developed using the DAB substrate kit (Vector Laboratories, Burlingame, CA, USA), dehydrated with ethanol, cleared with xylene, and mounted with Permount^®^ (Fisher, Pittsburg, CA, USA).

### 4.4. FXTAS Neuropathology Evaluation

We evaluated FXTAS neuropathology using the Diagnostic Criteria for the Neuropathological Assessment of FXTAS Disorder [23]. Standardized neuropathological examinations were carried out blind to any clinical data. H&E staining was used to semi-quantitatively classify neuropathological lesions of the hippocampus into four morphological states of severity: Normal (1): no damage; Mild (2): involved up to 39%; Moderate (3): involved 40–70%; Severe (4): more than 70% involvement (70 to 100%).

### 4.5. Quantification of Cells and Intranuclear Inclusion in the Hippocampus 

We assessed the number of neurons and astrocytes and the number and percentage of neurons and astrocytes with intranuclear inclusions in each hippocampal area of interest. The hippocampus has three distinct zones: (1) the hippocampus proper, i.e., cornu ammonis (CA), including CA1 to CA4, with pyramidal neurons; (2) the dentate gyrus (DG), with a granular layer; and (3) the subiculum. In this study, we considered CA1 and CA3 of the proper hippocampus and two areas of DG, which we called DG1 and DG2 (Figure 1A shows the four sub-regions).

Then, every neuron and astrocyte with and without inclusion within the ROIs were measured at 20× magnification using a Nikon E600 ECLIPSE. Quantification was also performed on three slides from each case. On H&E-stained sections, cell and inclusion identification within the ROIs was performed based on standard morphological criteria as previously described [23]. The neuron was demonstrated by its size, abundant cytoplasm, and large, centrally placed, round nucleus with a single prominent nucleolus. Astrocytes were diagnosed by their irregular, ovoid nuclei with clear euchromatin and the lack of eosinophilic cytoplasm and nucleoli. In addition to the mentioned criteria enabling us to distinguish neuronal and astrocytic nuclei, another helping feature is the location of astrocytes, whether perivascular or immediately adjacent to the cytoplasmic borders of neurons. Oligodendrocytes had small, round, basophilic, densely stained (hyperchromatic) nuclei and an absence of nucleoli or cytoplasm. The nuclei of microglia were easily identified with basophilic club-shaped terminations [23]. The number of (neuronal or astroglia) inclusions was indicated as a ratio of inclusion-bearing cells per total number of those cells (with and without inclusions) counted (i.e., number with inclusions/total sample). Then, the individual ratios were used to quantify the percentage of each cell type bearing inclusion. The number of neuronal or astroglia inclusions for each area was calculated, except for the neuronal inclusions in the DG1 and DG2 sub-regions.

### 4.6. Statistical Analysis

SPSS Statistics Version 21.0 was used for the analysis. The average data collected from the three slices per case analyzed were compared between FXTAS and the control groups using the non-parametric Mann–Whitney *U* test (two independent nonparametric analyses). To examine the relationships between CGG repeat size, age, cell number, and the number of cells with intranuclear inclusions, correlation analyses were performed using Pearson’s correlation. Pearman’s rank-order correlation was also employed to evaluate the associations of the pathology findings with molecular measures (e.g., number of CGG repeats), age, and gender. A significant value of *p* < 0.05 was set for all analyses. Quantifications were then compared to previously reported cases [23,27,28,29] (Table 4).

## 5. Conclusions

Neuropathology changes in the hippocampus with FXTAS comprise white matter disease, neuron loss, gliosis, and intranuclear inclusions in astrocytes and neurons. These changes were milder in females; however, the number of neurons and astrocytes with intranuclear inclusions was similar in males and females. Astrocytes had more and larger inclusions than neurons. We postulate that vacuolar degeneration in white matter and significant pyramidal neuronal loss contribute to hippocampal atrophy. Cases with an age of death greater than 60 presented a negative correlation between age of death and CGG repeat size. Nevertheless, there was not a significant correlation between the number of intranuclear inclusions and age at death. Generally, higher CGG repeat numbers were associated with hippocampal white matter change and neuron depletion, while the number of astrocytes and neurons bearing inclusions increased with elevated CGG repeat length. 

## Figures and Tables

**Table 1:** Case characteristics of FXTAS and control groups.

Case Number	Gender	Age	Blood CGG	PMI (h)	Cause of Death
Control 1	F	75	N/A	53	Cardiovascular disease
Control 2	F	69	N/A	60	Heart failure
Control 3	M	62	N/A	27	Sudden cardiac death (SCD)
Control 4	F	63	N/A	12	Hemorrhagic stroke
Control 5	F	86	N/A	61	Subdural hematoma
Control 6	M	60	N/A	22	Liver Failure
Control 7	F	76	N/A	5	Aspergillosis
Control 8	F	69	N/A	57	Sudden cardia death (SCD)
Control 9	M	53	N/A	45	Multi-organ dysfunction syndrome (MODS)
Case 1	M	79	88 or 81	7.25	-
Case 2	M	85	89	-	-
Case 3	F	66	30, 80	-	-
Case 4	M	53	57	-	-
Case 5	F	65	-	240	-
Case 6	M	69	98	6.5	Cardiac failure
Case 7	M	76	87 or 85	-	-
Case 8	M	-	89	20	-
Case 9	F	52	36, 75	-	Multiple Sclerosis
Case 10	F	86	29, 87	-	-
Case 11	M	72	-	13.5	Myelodysplasia
Case 12	M	85	66	62	-
Case 13	M	82	67	-	-
Case 14	M	82	91	-	-
Case 15	F	79	30, 78	-	Lung cancer
Case 16	F	80	30, 63	5	Natural causes
Case 17	M	67	112	-	-
Case 18	M	66	105	30	Cardiorespiratory arrest
Case 19	M	75	89 or 78	22	Lung cancer
Case 20	M	89	75	-	-
Case 21	M	77	71 or 77	-	-
Case 22	M	85	86	127	Cardiorespiratory arrest
Case 23	M	87	70	-	Stroke
Case 24	M	70	62, 75	2.5	-
Case 25	M	34	122	-	Gunshot
Case 26	-	-	-	-	-

**Table 2:** Number of total neurons and astrocytes in CA1&3 and DG1&2 areas of the control hippocampus.

Case	CA1 Neuron	CA3 Neuron	DG1 Neuron	DG2 Neuron	CA1 Astrocyte	CA3 Astrocyte	DG1 Astrocyte	DG2 Astrocyte
Control 1	130	86	82	163	34	39	27	21
Control 2	167	73	105	100	28	36	10	10
Control 3	96	49	41	82	32	43	22	10
Control 4	116	60	82	130	31	56	14	11
Control 5	63	47	108	119	-	31	8	-
Control 6	-	75	115	117	-	28	21	27
Control 7	72	53	107	113	61	31	18	12
Control 8	45	49	113	163	27	29	12	11
Control 9	54	42	123	-	15	27	-	-

“-” No information.

**Table 3:** Number of total neurons and astrocytes, and the number of neuronal and astrocytic inclusions (actual counts and percentages) in CA1&3 and DG1&2 of FXTAS hippocampus.

Case Number	CA1 Neurons Inclusion	% CA1 Neurons Inclusion	CA3 Neurons Inclusion	% CA3 Neurons Inclusion	CA1 Astrocyte Inclusion	% CA1 Astrocyte Inclusion	CA3 Astrocyte Inclusion	% CA3 Astrocyte Inclusion	DG1 Astrocyte Inclusion	% DG1 Astrocyte Inclusion	DG2 Astrocyte Inclusion	% DG2 Astrocyte Inclusion
Case 1	4/60	6.6	8/35	22.85	2/44	4.54	3/34	8.8	10/27	37	3/23	13
Case 2	2/36	5.5	18/75	24	3/38	7.89	1/36	2.7	6/17	35.2	7/27	25.9
Case 3	8/94	8.5	3/40	7.5	1/10	10	1/20	5	0/11	0	1/5	20
Case 4	0/57	0	0/46	0	0/11	0	0/64	0	0/13	0	0/11	0
Case 5	4/75	5.3	4/52	7.69	0/17	0	11/20	55	5/9	55.5	1/6	16.6
Case 6	4/50	8	5/33	15.15	2/13	15.38	5/18	27.7	7/22	31.8	5/11	45.45
Case 7	1/68	1.47	2/60	3.33	2/28	7.1	8/53	15	11/21	52.38	13/33	39.39
Case 8	4/55	7.27	1/34	2.94	0/39	0	4/42	9.5	9/25	36	2/17	11.76
Case 9	8/59	13.55	3/60	5	1/52	1.9	1/17	5.88	0/10	0	0/7	0
Case 10	6/50	12	1/32	3.12	0/48	0	0/23	0	2/12	16.6	2/10	20
Case 11	8/60	13.33	8/28	28.5	2/14	14.28	8/29	27.58	3/7	42.85	3/6	50
Case 12	2/90	2.22	0/33	0	0/12	0	0/33	0	0/17	0	0/16	0
Case 13	0/58	0	0/48	0	2/26	7.69	0/28	0	0/2	0	0/6	0
Case 14	4/51	7.84	12/85	14.12	0/22	0	6/49	12.24	7/15	46.6	2/9	22.2
Case 15	4/151	2.64	2/44	4.54	5/26	19.2	6/40	15	1/14	7.1	0/12	0
Case 16	0/85	0	0/87	0	0/25	0	0/42	0	0/9	0	0/11	0
Case 17	4/35	11.42	4/37	10.8	6/29	20.68	9/47	19	0/6	0	2/7	28.57
Case 18	2/47	4.25	-	-	0/14	0	-	-	6/11	54.5	6/19	31.57
Case 19	0/24	0	1/39	2.56	1/16	6.25	7/33	21.2	1/10	10	5/17	29.4
Case 20	6/22	27.27	4/59	6.78	5/12	41.6	1/35	2.85	5/19	26.3	3/10	30
Case 21	6/85	7.06	5/47	10.64	4/28	14.28	3/36	8.3	3/16	18.75	5/12	41.66
Case 22	1/36	2.77	4/47	8.5	7/25	28	5/35	14.2	6/11	54.5	1/11	9
Case 23	2/35	5.71	7/46	15.2	2/6	33.3	0/18	0	0/4	0	2/7	28.57
Case 24	3/57	5.26	5/33	15.15	0/24	0	5/38	13.1	1/10	10	1/12	8.3
Case 25	4/25	16	2/31	6.45	5/23	21.73	6/30	20	11/27	40.7	8/20	40
Case 26	2/52	3.84	6/55	10.9	4/13	30.76	5/42	11.9	10/22	45.4	4/14	28.57

“-” Not available. The number of inclusions were showed as ratios of (neurons and astrocytes) cells with inclusions per total number of cells counted, and as percentage (%) of cells (neurons and astrocytes) containing inclusions, within regions of interests (ROIs).

**Table 4:** Intranuclear inclusion counts in hippocampus with FXTAS.

	CA1 Neurons	CA1 Astroglia	CA3 Neurons	CA3 Astroglia	DG Neurons	DG Astroglia
Number (+)	83 of 1517	54 of 615	105 of 1186	95 of 862	-	180 of 706
Mean	5.47	8.78	8.85	11.02	-	25.49
Study 1 *	4.09%	4.49%	-	-	2.82%	6.35%
Study 2 *	10.1%	10.3%	-	-	2.1%	26.6%
Study 3 *	6.59%	8.84%	-	-	4.66%	11.56%
Study 4 *	-	-	-	-	0.68%	1.34%

Number of neurons and astrocytes with intranuclear inclusions in FXTAS cases of this study as a ratio of the number of cells counted (+) and mean, and also, intranuclear inclusion prevalence data previously reported. (“-”: Data not available. CA1 and CA3: pyramidal cell layers; DG: granule cell layer. * Study 1: Pretto et al., 2013 [29]; Study 2: Greco et al., 2006 [23]; Study 3: Tassone et al., 2012 [28]; Study 4: Hunsaker et al., 2011 [27].

## Data Availability

All data is available within the article and supplementary tables.

## Supplementary Materials

The supporting information can be downloaded at: https://www.mdpi.com/article/10.3390/ijms242417266/s1.
