# Supplementary material for: Neurodegeneration of White and Gray Matter in the Hippocampus with FXTAS
Source: Int J Mol Sci. 2023 Dec 8;24(24):17266. doi: 10.3390/ijms242417266 (PMC10743470; doi:10.3390/ijms242417266)
Supplement: Supplementary file 1 [file ijms-24-17266-s001.zip › ijms-2662019-supplementary.pdf]

**Table S1.** Macroscopic lesions in hippocampus of FXTAS cases

| Case Number | Macroscopic lesions |
|---|---|
| **Case 1** | Mild generalized atrophy and moderate dilation of lateral ventricles |
| **Case 2** | Unremarkable |
| **Case 4** | Mild increase in ventricular size and expansion of the cerebral white matter (edema) |
| **Case 5** | Decreased size of the hippocampus and marked diffuse ventriculomegaly |
| **Case 6** | White matter disease and mild ventricular dilation (MRI) |
| **Case 7** | Decreased size of the hippocampus and ventriculomegaly |
| **Case 9** | Moderate frontal gyral atrophy, multiple irregular soft regions of grayish discoloration – prominent in periventricular |
| **Case 10** | Left hemisphere shows marked decrease in ventricular size |
| **Case 12** | Marked decrease in the size of the amygdala and hippocampus, moderate ventricular dilation |
| **Case 13** | Moderate frontoparietal atrophy and ventricles decreased in size |
| **Case 14** | Mild frontal cortical atrophy |
| **Case 15** | Mild atrophy and scattered white matter disease (MRI) |
| **Case 16** | Moderate frontal cortical atrophy, moderate enlargement of ventricles, marked thinning of gray matter ribbon in all lobes to 2mm |
| **Case 18** | Diffuse frontoparietal and superior temporal gyral atrophy, mild edema, equivocal decrease in ventricular size |
| **Case 19** | Moderate frontoparietal and temporal lobe atrophy |
| **Case 21** | Atrophy of the rostral superior frontal gyrus, compressed ventricles, which are not clearly dilated, irregular small areas of grayish discoloration in white matter, which are not periventricular |
| **Case 22** | Mild frontal temporal-parietal atrophy, moderately dilated ventricles |

Not applicable for cases 3, 8, 11, 17, 20, and 23 to 26.

**Table S2.** Neuropathology in hippocampus in 26 FXTAS cases

| Case Number | Inclusions (neurons + astrocytes) | CA1 neuron cell drop | CA3 neuron cell drop | (S) | (G) | (PVC) | (Co) | (H) | (T) | (Cl) | (He) |
|---|---|---|---|---|---|---|---|---|---|---|---|
| **Case 1** | Yes | 3 | 3 | 2 | 2 | 3 | 3 | 2 | 2 | 1 | 1 |
| **Case 2** | Yes | 3 | 1 | 2 | 2 | 2 | 1 | 1 | 1 | 2 | 1 |
| **Case 3** | Yes | 1 | 2 | 1 | 2 | 2 | 3 | 1 | 1 | 2 | 2 |
| **Case 3** | No | 3 | 2 | 4 | 2 | 2 | 3 | 1 | 2 | 2 | 1 |
| **Case 4** | Yes | 2 | 2 | 1 | 4 | 1 | 2 | 1 | 2 | 2 | 1 |
| **Case 5** | Yes | 3 | 3 | 1 | 2 | 2 | 1 | 1 | 2 | 2 | 1 |
| **Case 6** | Yes | 2 | 1 | 1 | 2 | 2 | 1 | 1 | 1 | 1 | 2 |
| **Case 7** | Yes | 3 | 3 | 1 | 1 | 2 | 2 | 1 | 2 | 2 | 1 |
| **Case 8** | Yes | 3 | 1 | 2 | 2 | 2 | 2 | 1 | 2 | 2 | 1 |
| **Case 9** | Yes | 3 | 3 | 1 | 3 | 2 | 1 | 1 | 2 | 2 | 1 |
| **Case 11** | Yes | 3 | 3 | 1 | 2 | 2 | 3 | 2 | 2 | 3 | 2 |
| **Case 12** | Rare | 2 | 3 | 3 | 1 | 2 | 2 | 2 | 2 | 2 | 2 |
| **Case 13** | Rare | 3 | 2 | 1 | 3 | 3 | 3 | 2 | 2 | 2 | 2 |
| **Case 14** | Yes | 3 | 1 | 2 | 2 | 3 | 2 | 1 | 2 | 2 | 1 |
| **Case 15** | Yes | 1 | 2 | 2 | 2 | 2 | 3 | 2 | 3 | 2 | 2 |
| **Case 16** | No | 2 | 1 | 1 | 2 | 2 | 1 | 1 | 1 | 2 | 1 |
| **Case 17** | Yes | 3 | 3 | 1 | 1 | 2 | 1 | 1 | 1 | 1 | 1 |
| **Case 18** | Yes | 3 | - | 3 | 2 | 2 | 1 | 1 | 2 | 2 | 1 |
| **Case 19** | Yes | 4 | 3 | 1 | 3 | 2 | 2 | 2 | 2 | 3 | 2 |
| **Case 20** | Yes | 4 | 2 | 3 | 2 | 2 | 2 | 1 | 2 | 2 | 2 |
| **Case 21** | Yes | 2 | 2 | 2 | 2 | 3 | 3 | 3 | 2 | 2 | 1 |
| **Case 22** | Yes | 3 | 2 | 1 | 3 | 3 | 1 | 2 | 3 | 2 | 1 |
| **Case 23** | Yes | 3 | 2 | 1 | 2 | 2 | 2 | 1 | 1 | 2 | 1 |
| **Case 24** | Yes | 3 | 3 | 1 | 1 | 2 | 1 | 2 | 2 | 1 | 2 |
| **Case 25** | Yes | 4 | 3 | 1 | 2 | 1 | 1 | 1 | 2 | 1 | 1 |
| **Case 26** | Yes | 3 | 2 | 1 | 2 | 2 | 1 | 1 | 1 | 2 | 1 |

Spongiosis (S), Gliosis (G), Perivascular cuffing (PVC), Congestion (Co), Microhemorrhage (H), Vessel wall thickness (T), Clearing (Cl), Hemosiderin (He); All scales of severity: normal or minimal abnormality (1), mild (2), moderate (3), extensive/severe (4). NA, tissue not available for analysis, either because specific samples were unavailable or because fixation artefacts precluded further analysis. Reference for NIA-Reagan criteria (Newell et al., 1999).

**Table S3.** Additional significant histopathology in hippocampus of 26 FXTAS cases

| Case Number | Histopathology |
|---|---|
| **Case 1** | Atherosclerosis |
| **Case 3** | Neuropathology of Alzheimer's; no Lewy bodies; Lewy body disease |
| **Case 4** | Sever diffuse acute cerebral ischemic damage |
| **Case 5** | Scattered hypertrophic astrocytes with fibrillary processes, neurofibrillary tangles and beta-amyloid plaques |
| **Case 10** | Mild, patchy cerebrovascular aterosclerosis |
| **Case 12** | Diffuse, moderately severe ASD |
| **Case 13** | Tangles in hippocampus, no atherosclerosis |
| **Case 15** | Small vessel disease |
| **Case 16** | Pathologically met the criteria for Alzheimers; Prominent GVD and scattered plaques and tangles present in CA1 |
| **Case 17** | Minimal patchy athersclerosis |
| **Case 18** | GFAP stain shows scattered reactive astrocytes, with patchy densities in deep white matter |
| **Case 19** | CA1 shows diffuse brick-red change in the neurons; acute ischemic damage to hippocampus |
| **Case 20** | Intermediate stage alzheimer's disease |
| **Case 21** | Biels stain shows small number of neuritic plaques in gray matter corresponding to aging. Loss of axons was seen in patchy areas of pallor in white matter, which corresponded to myelin loss. LFB-PAS showed decreased number of glial cells in these abnormal areas, moderate spotty athersclerosis |
| **Case 22** | Scant granulovacuolar degeneration |

No additional or significant information for cases: 2, 6 to 9, 11, 14, 23 to 26.

**Table S4.** Number of total necrotic neurons (actual counts and percentages) in CA1&3 and DG1&2 subregions of FXTAS hippocampi.

| Case Number | nN CA1 | % nN CA1 | nN CA3 | % nN CA3 | nN DG1 | % nN DG1 | nN DG2 | % nN DG2 |
|---|---|---|---|---|---|---|---|---|
| **Case 1** | 1/60 | 1.5 | 1/35 | 2.85 | 0/67 | 0 | 1/90 | 0.9 |
| **Case 2** | 17/36 | 47.2 | 12/75 | 16 | 4/116 | 3.44 | 8/178 | 4.49 |
| **Case 3** | 0/94 | 0 | 3/40 | 7.5 | 0/28 | 0 | 1/122 | 0.8 |
| **Case 4** | 11/57 | 19.3 | 2/46 | 4.35 | 0/95 | 0 | 0/103 | 0 |
| **Case 5** | 4/75 | 5.3 | 6/52 | 11.53 | 1/95 | 1.05 | 3/169 | 1.77 |
| **Case 6** | 6/50 | 12 | 3/33 | 9.09 | 4/96 | 4.16 | 1/175 | 0.57 |
| **Case 7** | 3/68 | 4.4 | 5/60 | 8.33 | 4/106 | 3.77 | 2/102 | 1.96 |
| **Case 8** | 2/55 | 3.63 | 2/34 | 5.88 | 16/57 | 28.07 | 6/147 | 4.08 |
| **Case 9** | 3/59 | 5.08 | 5/60 | 8.33 | 0/139 | 0 | 0/99 | 0 |
| **Case 10** | 2/50 | 4 | 3/32 | 9.37 | 1/164 | 0.6 | 2/240 | 0.83 |
| **Case 11** | 2/60 | 3.33 | 7/28 | 25 | 0/105 | 0 | 3/78 | 3.85 |
| **Case 12** | 3/90 | 3.33 | 4/33 | 12.12 | 3/76 | 3.95 | 3/85 | 3.53 |
| **Case 13** | 0/58 | 0 | 1/48 | 2.08 | 2/65 | 3.07 | 1/128 | 0.78 |
| **Case 14** | 3/51 | 5.9 | 2/85 | 2.35 | 0/120 | 0 | 2/171 | 1.17 |
| **Case 15** | 13/151 | 8.6 | 7/44 | 15.9 | 8/118 | 6.77 | 2/51 | 3.92 |
| **Case 16** | 7/85 | 8.23 | 3/87 | 3.45 | 3/75 | 4 | 3/110 | 2.73 |
| **Case 17** | 1/35 | 2.8 | 0/37 | 0 | 1/149 | 0.67 | 2/124 | 1.6 |
| **Case 18** | 7/47 | 14.89 | - | - | 1/120 | 0.83 | 2/146 | 1.37 |
| **Case 19** | 4/24 | 16.6 | 0/39 | 0 | 1/59 | 1.69 | 2/218 | 0.92 |
| **Case 20** | 5/22 | 22.72 | 13/59 | 22.03 | 2/28 | 7.4 | 1/130 | 0.77 |
| **Case 21** | 6/85 | 7.06 | 3/47 | 6.38 | 3/175 | 1.7 | 16/180 | 8.88 |
| **Case 25** | 0/25 | 0 | 3/31 | 9.68 | 3/125 | 2.4 | 4/98 | 4.08 |
| **Case 26** | 8/52 | 15.38 | 6/55 | 10.9 | 2/112 | 1.78 | 3/96 | 3.13 |

The number of necrotic neurons were revealed as ratios of necrotic neurons per total number of neurons counted, and as percentage (%) of necrotic neurons. Necrotic neurons shown as nN CA1, nN CA3, nN DG1 and nN DG2; "-" Not available.
